# A Bayesian Model for the Prediction and Early Diagnosis of Alzheimer's Disease

**DOI:** 10.3389/fnagi.2017.00077

**Published:** 2017-03-31

**Authors:** Athanasios Alexiou, Vasileios D. Mantzavinos, Nigel H. Greig, Mohammad A. Kamal

**Affiliations:** ^1^Novel Global Community Educational FoundationalHebersham, NSW, Australia; ^2^Department of Computer Science and Biomedical Informatics, University of ThessalyLamia, Greece; ^3^Drug Design and Development Section, Translational Gerontology Branch, Intramural Research Program, National, Institute on Aging, National Institutes of Health, Biomedical Research CenterBaltimore, MD, USA; ^4^Metabolomics and Enzymology Unit, Fundamental and Applied Biology Group, King Fahd Medical Research Center, King Abdulaziz UniversityJeddah, Saudi Arabia; ^5^EnzymoicsHebersham, NSW, Australia

**Keywords:** Alzheimer's disease, early diagnosis, medical decision systems, Bayesian statistics, Markov Chain Monte Carlo, Metropolis-Hastings Algorithm, Gibbs Sampling, Winbugs

## Abstract

Alzheimer's disease treatment is still an open problem. The diversity of symptoms, the alterations in common pathophysiology, the existence of asymptomatic cases, the different types of sporadic and familial Alzheimer's and their relevance with other types of dementia and comorbidities, have already created a myth-fear against the leading disease of the twenty first century. Many failed latest clinical trials and novel medications have revealed the early diagnosis as the most critical treatment solution, even though scientists tested the amyloid hypothesis and few related drugs. Unfortunately, latest studies have indicated that the disease begins at the very young ages thus making it difficult to determine the right time of proper treatment. By taking into consideration all these multivariate aspects and unreliable factors against an appropriate treatment, we focused our research on a non-classic statistical evaluation of the most known and accepted Alzheimer's biomarkers. Therefore, in this paper, the code and few experimental results of a computational Bayesian tool have being reported, dedicated to the correlation and assessment of several Alzheimer's biomarkers to export a probabilistic medical prognostic process. This new statistical software is executable in the Bayesian software Winbugs, based on the latest Alzheimer's classification and the formulation of the known relative probabilities of the various biomarkers, correlated with Alzheimer's progression, through a set of discrete distributions. A user-friendly web page has been implemented for the supporting of medical doctors and researchers, to upload Alzheimer's tests and receive statistics on the occurrence of Alzheimer's disease development or presence, due to abnormal testing in one or more biomarkers.

## Introduction

**Figure 1:**
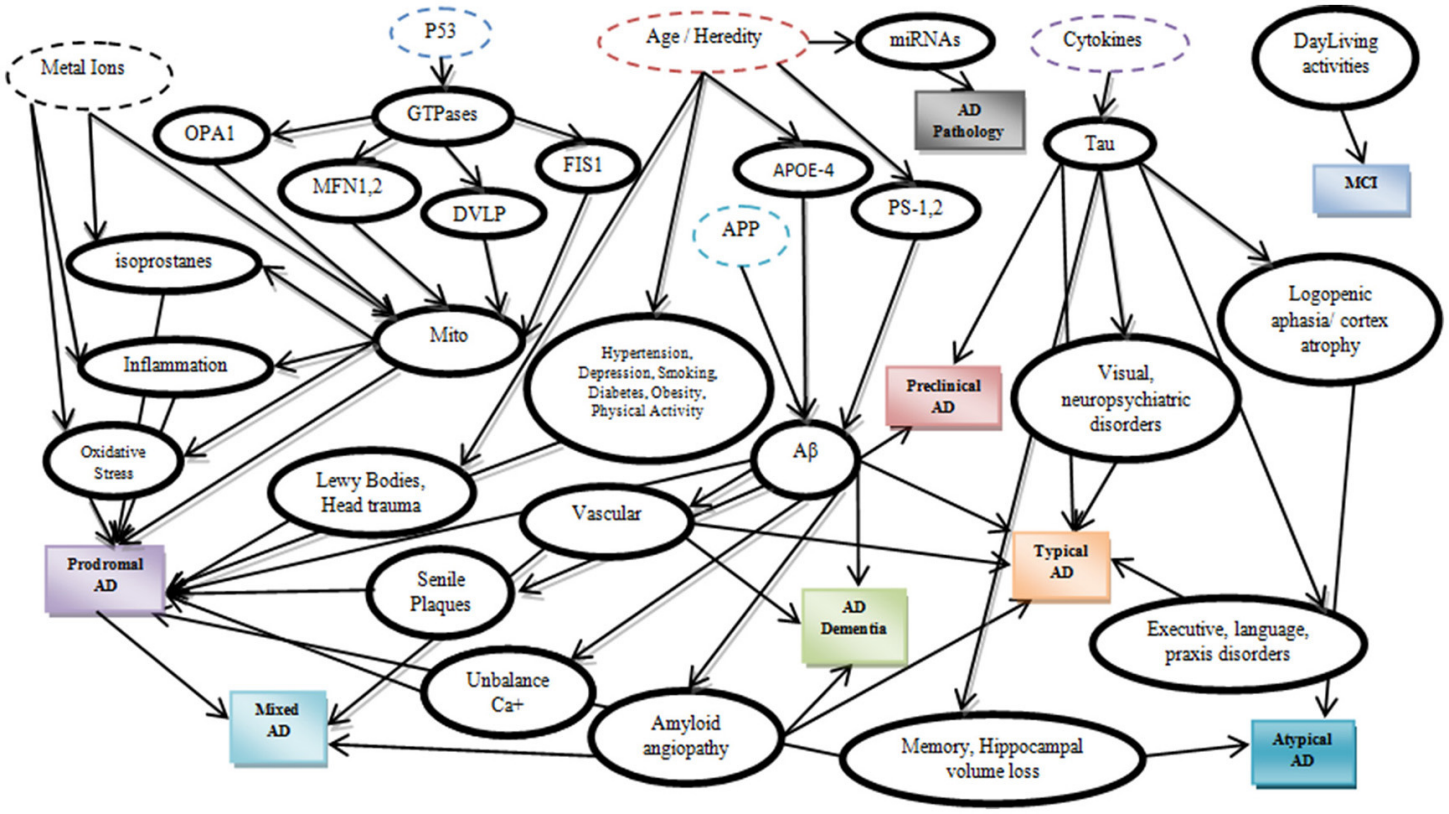
**Alzheimer's disease biomarkers expressed through a Bayesian Network**.

A precise etiology of Alzheimer's disease (AD) is still unclear while several risk factors have been recognized to catalytically affect the early onset and the progression of the disease (Abbott and Dolgin, 2016). According to latest studies (Dubois et al., 2007), AD can be categorized according to potential risk factors, symptoms and pathophysiological lesions into eight different categories (Table 1). Furthermore, these eight categories can be analyzed in depth by adding potential biomarkers in each category (Figure 1) which have been proved to affect the severity of the disease (Mantzavinosa et al., 2017). While several attempts at reducing AD severity have already been presented targeting mainly the symptomatic treatment (Ashraf et al., 2015) until now, there is no holistic therapy available that can efficiently reverse AD. For many scientists and pharmaceuticals companies, there are several and different treatment approaches for AD such as cholinesterase inhibitors, NMDA receptor antagonist, β-secretase inhibitors, γ-secretase inhibitors, α-secretase stimulators, tau inhibitors, immunotherapy, nutraceuticals, and nano drugs (Ashraf et al., 2015; Soursou et al., 2015) even though the more secure solution seems to be the early diagnosis of neurodegeneration signs, in order to facilitate the early diagnosis or prediction.

**Table 1:** **Alzheimer's disease classification according to symptoms and lesions based on the “Research criteria for the diagnosis of Alzheimer's disease: revising the NINCDS-ADRDA criteria” (Abbott and Dolgin, 2016)**.

**Categories**	**Description**
Prodromal AD (Category1)	Clinical Symptoms, memory disorders, Hippocampal volume loss and biomarkers of CSF that lead to AD pathology
AD dementia (Category2)	The social function, the composite activities of the daily life are obstructed. This state is the threshold between memory changes and in one more cognitive factor
Typical AD (Category3)	Progressive memory loss, cognitive disorders, and neuropsychiatric modifications
Atypical AD (Category4)	Progressive aphasia, Logopenic aphasia, frontal AD morphology and cortical atrophy at the posterior section. Also, is supported from amyloidosis biomarkers in brain or CSF
Mixed AD (Category5)	Incidents that validate the diagnostic AD requirements for typical AD and there are disorders such as cerebrovascular disease or Lewy Bodies disease
Preclinical states of AD (Category6)	This state includes an *in vivo* amyloidosis evidence of the brain, or individuals whose families have the autosomal dominant mutation of AD
Alzheimer's Pathology (Category7)	Senile Plaques and Neurofibrillary tangles, loss of neuronal synapses, amyloid deficits in the cerebral vascular cortex
Mild cognitive impairment (Category8)	Individuals that abstain from the clinic biological character of AD and also have measurable MCI. Those individuals may suffer from AD, but there is no evidence for AD

In this regards, Bayesian Statistics constitutes a powerful tool for Science and especially for Biomedical Informatics and Medical Decision Systems. Markov Chain Monte Carlo (MCMC) theory was provided as a solution several times, targeting environmental' s or diseases' evaluations with satisfactory results (Tzoufras, 2009). Bayesian statistics uses all the unknown parameters as random variables, to pre-define the prior distribution of the model and calculate the posterior distribution f(θ|y), which can be expressed as:

$$\begin{array}{l} f\left(\theta |y\right)=\frac{f\left(y|\theta \right)f\left(\theta \right)}{f\left(y\right)}\propto f\left(y|\theta \right)f\left(\theta \right), \end{array}$$

or including both the prior and the observed data by the expression of the prior distribution f(θ) and the likelihood f(y|θ) as follows:

$$\begin{array}{l} f\left(y|\theta \right)=\prod_{i=1}^{n}f\left(y_{i}|\theta \right). \end{array}$$

In this research paper, a new probabilistic model was created, describing the relationship between AD biomarkers, which may reveal and influence the disease's development, presence or progression. The algorithmic approach to AD prediction coded with WinBUGS biostatistics software (Lunn et al., 2000) for Bayesian inference, data analysis, and modeling. The model, the initial data and few examples are described in the Experimental section of this paper.

## Materials and methods

### A probabilistic approach to AD

Let us recall some basic mathematical notations concerning the Bayesian approach (Congdon, 2005; Vidakovic, 2011; Højsgaard, 2012). Assume a random variable Y known as a response, which follows a probabilistic path f(y|θ), where θ is a parameter vector. We consider a sample y = [y_1_, y_2_,….,y_*n*_] of size n. If we assume two possible events A, B where A = A_1_ ∪ A_2_ ∪.…∪ A_n_, A_i_ ∩ A_j_ = ∅ ∀ i ≠ j, Bayes Theorem calculates the probability to occur an event A_*i*_ given B,

$$\begin{array}{l} \left(Ai|B\right)=\;\frac{P\left(B|Ai\right)P\left(Ai\right)}{P\left(B\right)}=\frac{P\left(B|Ai\right)P\left(Ai\right)}{\sum_{i=1}^{n}P\left(B|Ai\right)P\left(Ai\right)}. \end{array}$$

In general,

$$\begin{array}{l} P\left(A|B\right)=\frac{P\left(B|A\right)P\left(A\right)}{P\left(B\right)}\;\propto P\left(B|A\right)P\left(A\right). \end{array}$$

Finally, given the observed data y_1_, y_2_,…,y_n_, the posterior distribution f(θ|y_1_,…,y_n_) could be calculated from the prior distribution. Bayesian Inference is based on the p(θ|y) factor which is used by MCMC methods. Markov Chain Monte Carlo methods are based on iterative sampling from the posterior distribution, using various chain probabilities of the sample parameters and resulting posterior means and variances of the parameters or functions of the parameters Δ = Δ(θ) as follows:

$$\begin{array}{lll} E\left(\theta_{k}|y\right) & = & \int \theta_{k}p\left(\theta |y\right)d\theta ,\\ Var\left(\theta_{k}|y\right) & = & \int \theta_{k}^{2}p\left(\theta |y\right)d\theta -{\left[E\left(\theta_{\kappa }|y\right)\right]}^{2}=E\left(\theta_{k}^{2}|y\right)-{\left[E\left(\theta_{k}|y\right)\right]}^{2}, \end{array}$$

$$\begin{array}{lll} E\left[\Delta \left(\theta \right)|y\right] & = & \int \Delta \left(\theta \right)p\left(\theta |y\right)d\theta ,\\ Var\left[\Delta \left(\theta \right)|y\right] & = & \int \Delta^{2}p\left(\theta |y\right)d\theta -{\left[E\left(\Delta |y\right)\right]}^{2}\\ = & E\left(\Delta^{2}|y\right)-{\left[E\left(\Delta |y\right)\right]}^{2}. \end{array}$$

The most popular MCMC methods are the Metropolis-Hastings Algorithm (Metropolis et al., 1953; Hastings, 1970) and its particular case, the Gibbs Sampling (Geman and Geman, 1984). In 1988, Lauritzen and Spiegelhalter presented for the first time a Bayesian expert system, the “ASIA model,” introducing a fictitious medical decision system for the explanation of dyspnea due to a patient's recent visit to Asia and the presence of several other symptoms (Lauritzen and Spiegelhalter, 1988).

The proposed in this paper AD prediction model was established based on the Bayesian Networks (BN). According to BN theory, if we assume a directed graph G with N nodes, each node *n* ∈*N* has a number of paternal nodes pa(n) that may be linked with “child” nodes and the joint distribution for such a network given as follows:

$$\begin{array}{l} P\left(N\right)=\prod_{n\in N}p\left(n|pa\left(n\right)\right). \end{array}$$

By taking into consideration the latest calculations for the relative probabilities of AD progression due to certain brain lesions (Table 2) (Christen, 2000; de la Torre, 2002; Praticò et al., 2002; Modrego and Ferrández, 2004; Hooper et al., 2007; Cheung et al., 2008; Stone, 2008; Schuff et al., 2009; Snider et al., 2009; Wang et al., 2009; Israeli-Korn et al., 2010; Barnes and Yaffe, 2011; Nazem and Mansoori, 2011; Serrano-Pozo et al., 2011; Bird, 2012; Alzheimer's Association, 2015; Chakrabarty et al., 2015) and the majority of the published AD biomarkers (Albert et al., 2010, 2011; Besson et al., 2015; Cabezas-Opazo et al., 2015; Dong et al., 2015; Duce et al., 2015; Eskildsen et al., 2015; Jansen et al., 2015; Madeira et al., 2015; Michel, 2015; Nakanishi et al., 2015; Ossenkoppele et al., 2015; Østergaard et al., 2015; Quiroz et al., 2015; Ringman et al., 2015; Risacher et al., 2015; Sastre et al., 2015; Schindler and Fagan, 2015; Sutphen et al., 2015; Thordardottir et al., 2015; Cauwenberghe et al., 2016; Counts et al., 2016; Gaël et al., 2016; Yang et al., 2016) or calculating indirectly the relative probabilities, we designed a Bayesian model for the prediction of AD based on the abnormal testing of one or more biomarkers. The described probabilities were exported through major clinical trials globally and are continuously subject to updating and redefinition. The proposed model includes the main AD categories formulated by the categorical prior distribution.

$$\begin{array}{l} r\sim dcat\left(p\left[\right]\right), \end{array}$$

the majority of biomarkers that underlie AD severity and are represented as an acyclic graph.

**Table 2:** **Alzheimer's disease biomarkers, biomarkers' probabilistic impact on Alzheimer's disease presence and the corresponding bibliographic reference**.

**Biomarker**	**Relative probability related to AD progression**	**References**
Age (>85)	38%	Alzheimer's Association, 2015
Age (75–84)	43%	Alzheimer's Association, 2015
Age (65–74)	15%	Alzheimer's Association, 2015
Age (<65)	4%	Alzheimer's Association, 2015
Lewy Body disease	10-20% The only way to conclusively diagnose the Dementia with Lewy Bodies is through a postmortem autopsy, and it is quite difficult to be recognized as no Alzheimer's Disease	Alzheimer's Association, 2015
APP	10%,15%,50%	Bird, 2012
Hypertension	20%	Israeli-Korn et al., 2010
GTPases	<1%	Alzheimer's Association, 2015
Depression	13.2%	Modrego and Ferrández, 2004; Barnes and Yaffe, 2011
Smoking	27.4%	Barnes and Yaffe, 2011
Diabetes	6.4%	Barnes and Yaffe, 2011
Obesity	3.4%	Barnes and Yaffe, 2011
Physical Activity	17.7%	Barnes and Yaffe, 2011
APOE4	30-70%	Bird, 2012
PS 1,2	5%	Bird, 2012
Amyloid Angiopathy	80%	Serrano-Pozo et al., 2011
Oxidative Stress	25-30%	Christen, 2000
Inflammation	30-40%	de la Torre, 2002
Isoprostanes	50%	Praticò et al., 2002
P53	75%	Hooper et al., 2007
Cytokines	50%	Chakrabarty et al., 2015
miRNAs	60%	Wang et al., 2009
DVLP	74.3%	Wang et al., 2009
OPA1	61.4%	Wang et al., 2009
MFN1	27.8%	Wang et al., 2009
MFN2	33.6%	Wang et al., 2009
FIS1	60%	Wang et al., 2009
Visual, neuropsychiatric disorders	5%	Alzheimer's Association, 2015
Executive, language, praxis disorders	40%	Alzheimer's Association, 2015
DayLiving disorders	10-20%	Alzheimer's Association, 2015
Metal Ions	24%	Nazem and Mansoori, 2011
Unbalance Ca	5%	Shilling et al., 2014
Senile plaques	Over 60% until the Age of 80 and increases linearly on the Age	Stone, 2008
Amyloid Beta	Over 50% in Ages>85	Snider et al., 2009
Hippocampal volume loss/Memory Impairment	Approximately 10% of elders over the age of 70 years have significant memory loss and more than half of these individuals have AD	Schuff et al., 2009

**Figure 2:**
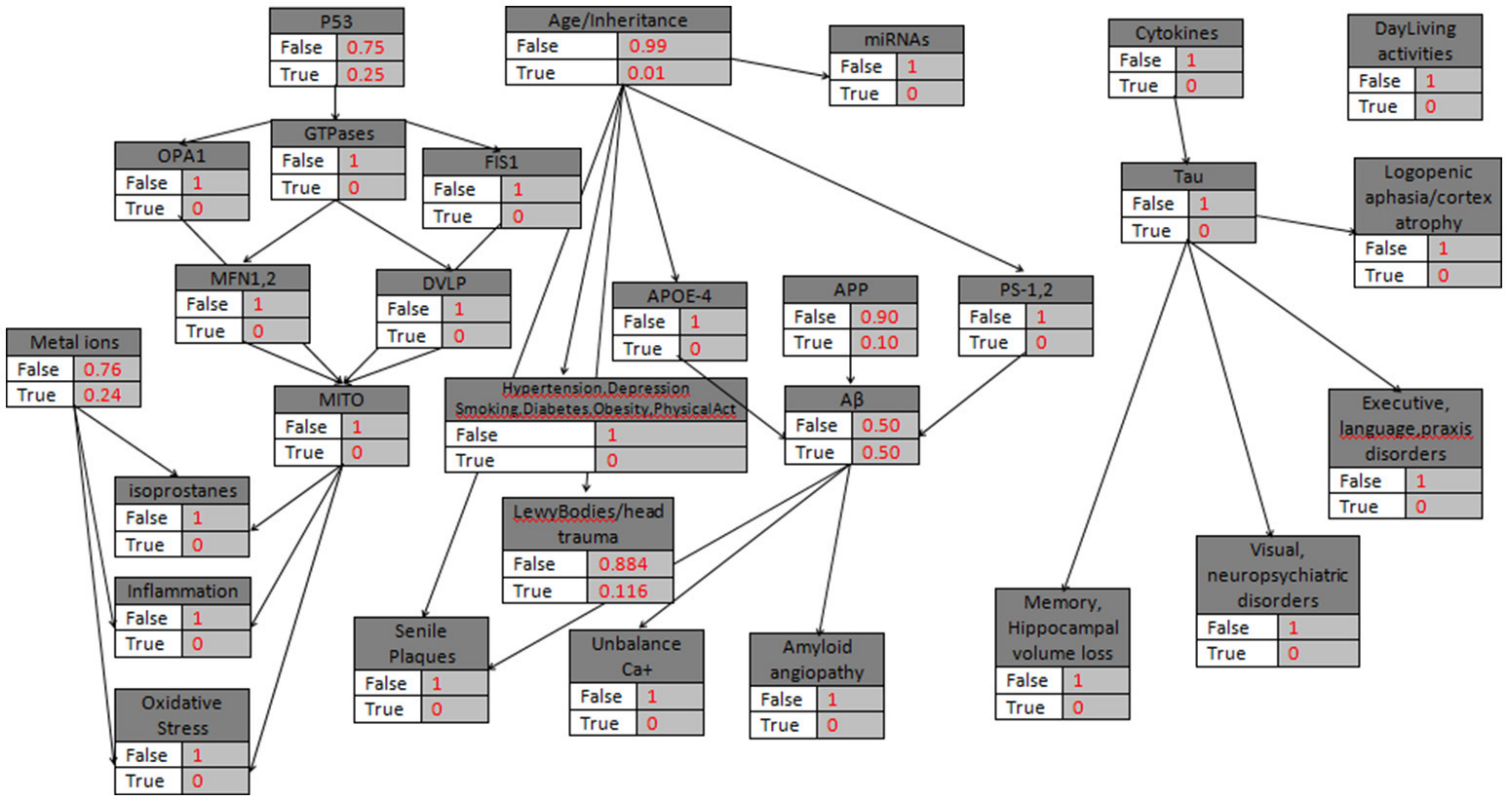
**The general probabilistic model with the knots initializations**. APP is set to 10%, Age>85, the “parent” knots and the LewyBodies are set to their probabilistic values.

**Figure 3:**
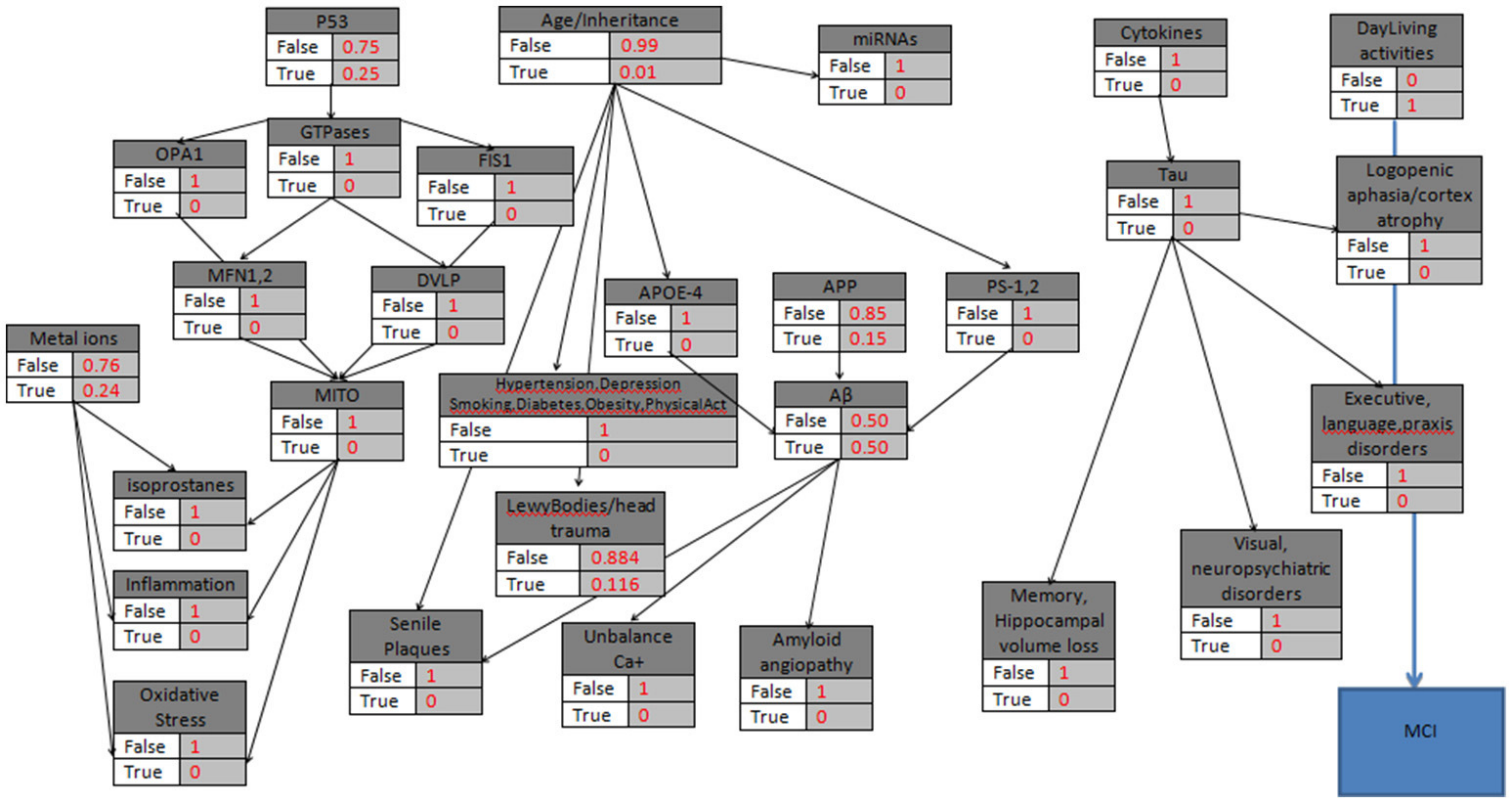
**The probabilistic model that can be used for MCI validation with the knots initializations**. APP is set to 15%, Age>85, the “parent” knots and the LewyBodies are set to their probabilistic values, and the DailyAcivities have a “strong” probability equal to 1.

**Figure 4:**
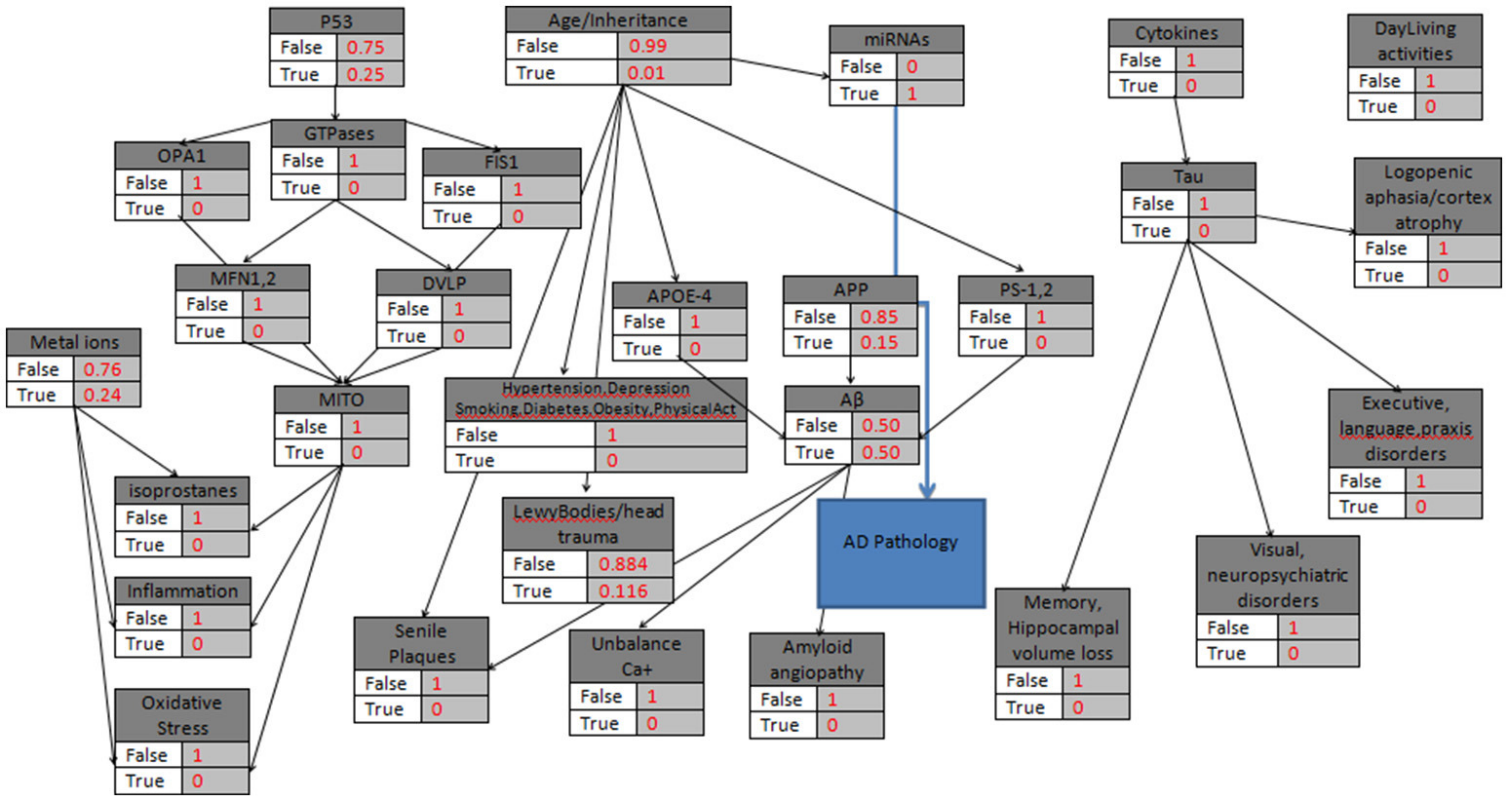
**The probabilistic model that can be used for AD Pathology validation with the knots initializations**. APP is set to 15%, Age>85, the “parent” knots and the LewyBodies are set to their probabilistic values, and the miRNAs have a “strong” probability equal to 1.

**Figure 5:**
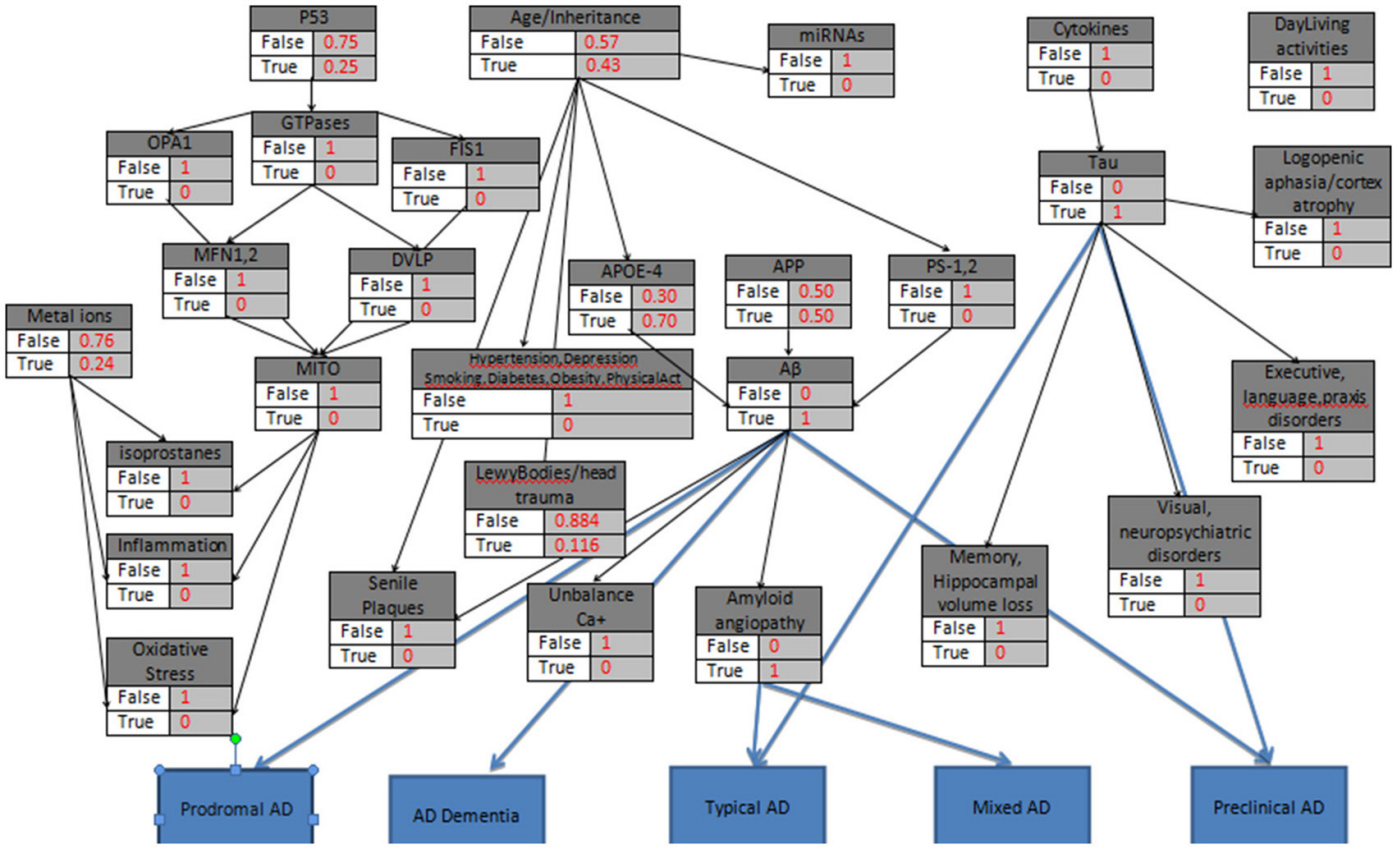
**The probabilistic model referring to several categories of Alzheimer's disease simultaneously, with the knots initializations**. APP is set to 50%, Age <60, the “parent” knots and the LewyBodies are set to their probabilistic values, and the biomarkers Tau, Aβ, APOE4, Amyloid Angiopathy have a “strong” probability equal to 1.

**Figure 6:**
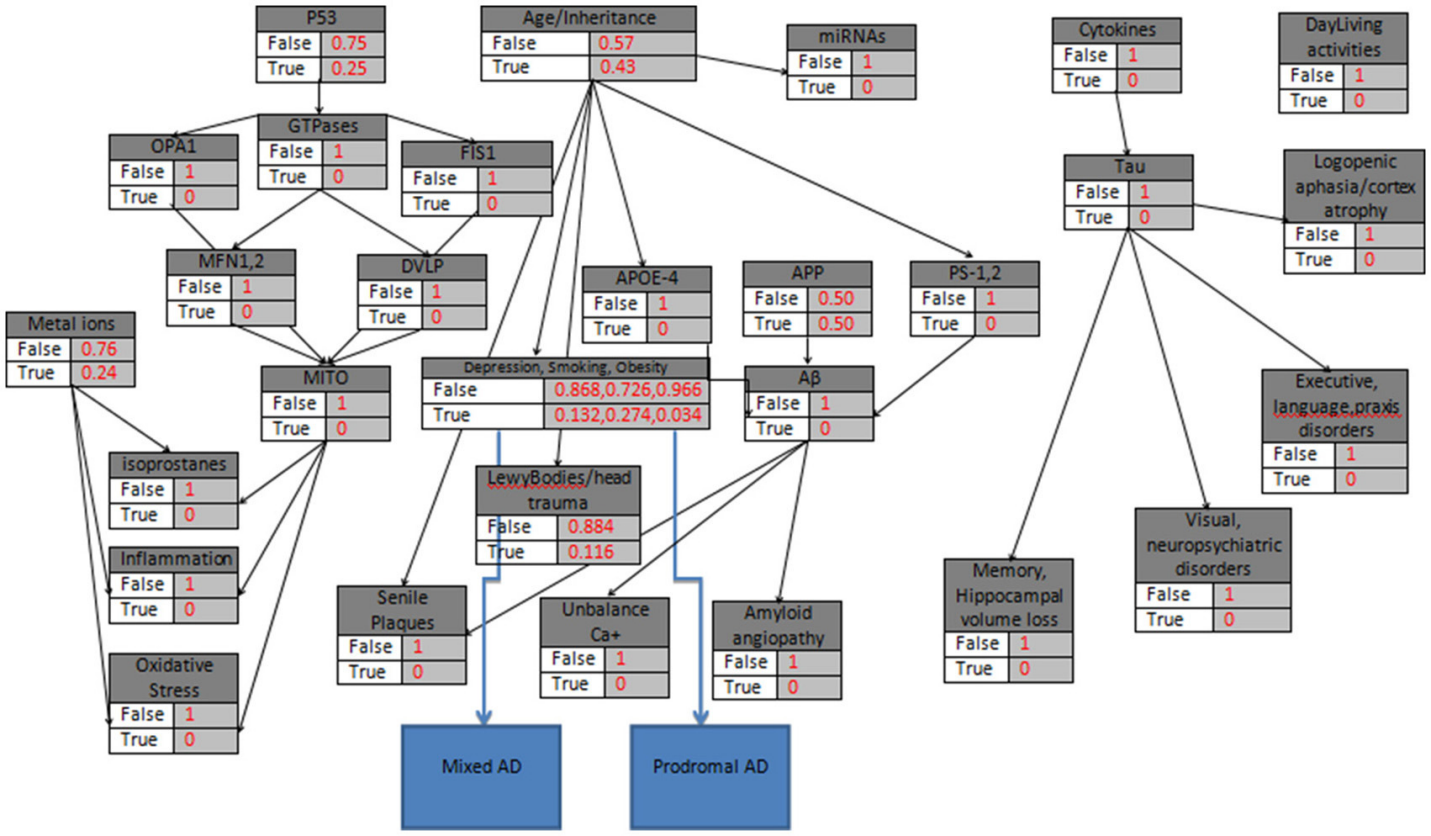
**The probabilistic model that can be used for Prodromal AD and Mixed AD validation due to Depression, Obesity and Smoking, with the knots initializations**. APP is set to 50%, Age <60, the “parent” knots, the LewyBodies and the Depression, Obesity and Smoking Biomarkers are set to their probabilistic values.

The Winbugs software requires all the parent knots of the acyclic graph to be initialized as True, something that does not affect the model execution. In the second step of the initialization mode, the “parent” knots Metal_Ions, p53, Age/Heredity, APP, Cytokines are defined with their probabilistic values that indicate the True value, and then all the “child” knots are simply set to False/True. An exception is proposed and occur in the case of LewyBodies existence, while the only way to conclusively diagnose the Dementia with Lewy bodies is through a postmortem autopsy and it is quite difficult to be recognized as a no Alzheimer's Disease case (Figure 2). When a biomarker is finally selected as True, then the probabilistic impact value is attributed to the related knot, according to Table 2 and the following rule: for the “parent” knots first we assign the probability to be False and then the probability to be True. For the “child” knots we assign probabilities in the form of False|False, False|True, True|False, True|True (Figures 3–**6**).

### Experimental

While a single biomarker can be related to more than one AD types, the probabilistic model consists of categorical variables-nodes (~dcat) where each variable node can be linked with two or more parent variables-nodes or can be presented as a single and independent variable-node. In the case where a node is linked to more than two parent nodes, another similar variable-node is created at the same level within the model. The proposed BN has been designed according to the latest “Research criteria for the diagnosis of Alzheimer's disease: revising the NINCDS-ADRDA criteria” (Dubois et al., 2007) and the model exports for every AD category the maximum probability value given by the biomarkers' evaluation, as it is described below along with the lists of initial values and data from the Winbugs Software.

**The General Form of the Model{**

Age~ dcat([1:2])

Ab~dcat(APOE4, PS1-2, APP[1:2])

Tau, Phospho ~ dcat(Cytokines 1:2])

MetalIons ~ dcat([1:2])

LewyBodies ~dcat(Age[1:2])

Hypertension ~ dcat(p.Hypertension[Age_Inheritance,1:2])

Depression ~ dcat(p.Depression[Age_Inheritance,1:2])

Smoking ~dcat(p.Smoking[Age_Inheritance,1:2])

Diabetes ~dcat(p.Diabetes[Age_Inheritance,1:2])

Obesity~dcat(p.Obesity[Age_Inheritance,1:2])

PhysicalActivity~dcat(p.PhysicalActivity[Age_Inheritance,1:2])

APP ~ dcat([1:2])

GTP ~ dcat(p53[1:2])

APOE4 ~ dcat(Age[1:2])

PS1-2 ~ dcat(Age[1:2])

Cytokines ~ dcat([1:2])

SenilePlaques ~ dcat(Ab[1:2])

UnbalanceCa ~ dcat(Ab[1:2])

Vascular ~ dcat(Ab, Tau_Phospho[1:2])

LogopenicAphasia, CortexAtrophy~dcat(Tau_Phospho[1:2])

Memory, HippocampalLoss~dcat(Tau, Phospho[1:2])

ExecLangPrax~dcat(Tau_Phospho[1:2])

Visual, Neuropsychiatric~dcat(Tau_Phospho[1:2])

DailyActivities ~ dcat([1:2])

OxidStress, Inflamation, Isoprostanes ~dcat( Mito, MetalIons [1:2])

Mito ~dcat( MetalIons,OPA1, MFN1,DVLP, FIS1 [1:2] )

MFN1~dcat(GTP[1:2])

OPA1~dcat(GTP[1:2])

DVLP~dcat(GTP[1:2])

FIS1~dcat GTP[1:2])

p53 ~ dcat([1:2])

miRNAs~dcat(Age[1:2])

MCI~dcat(DailyAct[1:2])

max1 ← max(Ab, LewyBodies, Mito, OxidStress,

   Memory_Hippocampal_loss, SenilePlaques,

   Unbalance_Ca, Hypertension_depression, Inflamation, Isoprostanes, Mito )

ProdromalAD ← max(max1,OxidStress)

ADdementia ← max(Ab, Vascular)

max2 ← max(Ab, Tau, Phospho, Vascular, ExecLangPrax)

TypicalAD ← max(max2, Visual, Neuropsychiatric)

AtypicalAD ← max(LogopenicAphasia, CortexAtrophy, Memory, HippocampalLoss)

MixedAD ← max(Vascular,Category1)

PreclinicalAD ← max(Ab, Tau, Phosph)

ADPathology ← miRNAs

MildCognitiveImpairment ← MCI

}

The model can be extended or adjusted to new biomarkers or relations between the symptoms, the lesions and the exported AD categories. Additionally, the relative probabilities can be updated or even more replaced by the biomarkers values when a secure protocol for AD diagnosis will be verified or proposed by the international health associations. Four examples are provided below concerning cases of abnormal biomarkers tests, revealing potential AD presence.

## Results

### Example 1

In the first hypothetical case study, a patient is assumed to be diagnosed with problems in daily living activities but with no other results of abnormal AD biomarkers. Additionally, the patient belongs to a risk group due to the age factor (>85). Therefore, while there is evidence only for abnormal Daily-Living activities, the corresponding node becomes “True,” and all the other nodes take the “False” value (Figure 3). The model calculates the *P*(*MCI*|*DailyLivingActivities*), the probability that Mild Cognitive Impairment is characterized ‘True’ given the DailyLivingActivities variable, which can be written as follows:

$$\begin{array}{l} P\left(MCI|DailyLivingActivities\right)\\ =\frac{P\left(MCI|DailyLivingActivities\right)P\left(MCI\right)}{P\left(DailyLivingActivities\right)}\\ P\left(MCI|DailyLivingActivities\right)=0.999. \end{array}$$

**Data List**

(Age_Inheritance =2, MetalIons=2, APP=2, Cytokines=2, DailyActivities=2, p53 = 2,

p.Age_Inheritance = c(0.99,0.01),

p.Ab = structure(.Data = c(0.50,0.50,0.50,0.50,0.50,0.50,0.50,0.50), .Dim = c(2,2,2)),

p.Tau_Phospho = structure(.Data = c(1,0,1,0), .Dim = c(2,2)),

p.MetalIons = c(0.76, 0.24),

p.LewyBodies = structure(.Data = c(0.884,0.116,0.884,0.116), .Dim = c(2,2)),

p.Hypertension = structure(.Data = c(1,0,1,0), .Dim = c(2,2)),

p.Depression = structure(.Data = c(1,0,1,0), .Dim = c(2,2)),

p.Smoking = structure(.Data = c(1,0,1,0), .Dim = c(2,2)),

p.Diabetes = structure(.Data = c(1,0,1,0), .Dim = c(2,2)),

p.Obesity = structure(.Data = c(1,0,1,0), .Dim = c(2,2)),

p.PhysicalActivity = structure(.Data = c(1,0,1,0), .Dim = c(2,2)),

p.APP = c(0.90,0.10),

p.GTP = structure(.Data = c(1,0,1,0), .Dim = c(2,2)),

p.APOE4 = structure(.Data = c(1,0,1,0), .Dim = c(2,2)),

p.PS1_2 = structure(.Data = c(1,0,1,0), .Dim = c(2,2)),

p.Cytokines = c(1,0),

p.SenilePlaques = structure(.Data = c(1,0,1,0,1,0,1,0), .Dim = c(2,2,2)),

p.Unbalance_Ca = structure(.Data = c(1,0,1,0,1,0,1,0), .Dim = c(2,2,2)),

p.Vascular = structure(.Data = c(1,0,1,0,1,0,1,0), .Dim = c(2,2,2)),

p.LogopenicAphasiaCortexAtrophy =structure(.Data = c(1,0,1,0), .Dim = c(2,2)),

p.MemoryHippocampalLoss = structure(.Data = c(1,0,1,0), .Dim = c(2,2)),

p.ExecLangPrax = structure(.Data = c(1,0,1,0), .Dim = c(2,2)),

p.VisualNeuropsychiatric =structure(.Data = c(1,0,1,0), .Dim = c(2,2)),

p.DailyActivities = c(0,1),

p.OxidStress1=structure(.Data = c(1,0,1,0,1,0,1,0), .Dim = c(2,2,2)),

p.OxidStress2=structure(.Data = c(1,0,1,0,1,0,1,0), .Dim = c(2,2,2)),

p.Inflamation1=structure(.Data = c(1,0,1,0,1,0,1,0), .Dim = c(2,2,2)),

p.Inflamation2=structure(.Data = c(1,0,1,0,1,0,1,0), .Dim = c(2,2,2)),

p.Isoprostanes1=structure(.Data = c(1,0,1,0,1,0,1,0), .Dim = c(2,2,2)),

p.Isoprostanes2=structure(.Data = c(1,0,1,0,1,0,1,0), .Dim = c(2,2,2)),

p.Mito1=structure(.Data = c(1,0,1,0,1,0,1,0), .Dim = c(2,2,2)),

p.Mito2=structure(.Data = c(1,0,1,0,1,0,1,0), .Dim = c(2,2,2)),

p.Mito3=structure(.Data = c(1,0,1,0), .Dim = c(2,2)),

p.MFN1 = structure(.Data = c(1,0,1,0), .Dim = c(2,2)),

p.OPA1= structure(.Data = c(1,0,1,0), .Dim = c(2,2)),

p.DVLP= structure(.Data = c(1,0,1,0), .Dim = c(2,2)),

p.FIS1= structure(.Data = c(1,0,1,0), .Dim = c(2,2)),

p.p53 = c(0.75,0.25),

p.Ab_APP= structure(.Data = c(1,0,1,0), .Dim = c(2,2)),

p.miRNAs=structure(.Data=c(1,0,1,0), .Dim = c(2,2)),

p.MCI_due_to_DayLiving=structure(.Data=c(0.001,0.999,0.001,0.999), .Dim = c(2,2)))

Executing the Winbugs code, the result for MCI category is the same as calculated above.

For each stochastic variable of the generated probabilistic model, Winbugs defines the categorical interval (Dubois et al., 2007; Abbott and Dolgin, 2016) for the categorical distribution ~dcat, which receives only positive values. The MCMC results, posterior summary estimations, mean, standard deviation and the estimation of the error is implemented by the batch mean method (Tables 3, 4). After 3000 and 10000 iterations of the current MCMC Winbugs algorithms, the mean value of MCI category can be similarly calculated as:

**Table 3:** **WINBUGS statistics for Alzheimer's disease categories according to Example 1**.

**Node**	**Mean (After 3000 Iterations)**	**Mean (After 10000 Iterations)**	**Standard deviation (After 3000 Iterations)**	**Standard deviation (After 10000 Iterations)**	**MC error (After 3000 Iterations)**	**MC error (After 10000 Iterations)**
Prodromal AD	1.566	1.562	0.4957	0.4961	0.007927	0.00473
AD dementia	1.506	1.502	0.5	0.5	0.008313	0.00458
Typical AD	1.506	1.502	0.5	0.5	0.008313	0.00458
Atypical AD	1.0	1.0	0.0	0.0	1.826E-12	1.0E-12
Mixed AD	1.566	1.562	0.4957	0.4961	0.007927	0.00473
Preclinical states of AD	1.506	1.502	0.5	0.5	0.008313	0.00458
Alzheimer's Pathology	1.0	1.0	0.0	0.0	1.826E-12	1.0E-12
Mild Cognitive Impairment	1.999	1.999	0.03649	0.03603	6.423E-4	3.667E-4

**Table 4:** **The total probability value for Alzheimer's disease presence due to alterations in DayLiving Activities**.

**Alzheimer's disease classification**	**Probability of Alzheimer's disease presence (in response to DayLiving Activities biomarker, after 3000 Iterations)**	**Probability of Alzheimer's disease presence (in response to DayLiving Activities biomarker, after 10000 Iterations)**
Prodromal AD	0.566	0.562
AD dementia	0.506	0.502
Typical AD	0.506	0.502
Atypical AD	0.0	0.0
Mixed AD	0.566	0.562
Preclinical states of AD	0.506	0.502
Alzheimer's Pathology	0.0	0.0
Mild Cognitive Impairment	0.999	0.999

*The results revealed the highest probability 0.999 for the case of Mild Cognitive Impairment, while Prodromal AD and Mixed AD show also high scores*.

$$\begin{array}{lll} EMCI & = & 2^{*}p_{MCI}+1.\;\left(1-p_{MCI}\right)\\ = & 2\;P\left(MCI|DailyLivingActivities\right)\\ = & p_{MCI}=2-1.999=0.999. \end{array}$$

### Example 2

In a similar case (age>85) where miRNAs' biomarker is assumed to be “True”, and there is no other evidence of heredity concerning AD (Figure 4), the model calculates the *P*(*ADPathology*|*miRNAs*). However, while miRNAs' node is also linked to the Age/Heredity node, there is a probabilistic relation between the Age/Heredity and miRNAs' nodes (Tables 5, 6).

$$\begin{array}{l} P\left(ADPathology|miRNAs\right)\\ =\frac{P\left(ADPathology|miRNAs\right)P\left(ADPathology\right)}{P\left(miRNAs\right)}\\ P\left(ADPathology|miRNAs\right)=1.0. \end{array}$$

**Table 5:** **WINBUGS statistics for Alzheimer's disease categories according to Example 2**.

**Node**	**Mean**	**Standard deviation**	**MC error after 10000 iterations in WinBugs**
Prodromal AD	1.562	0.4961	0.00473
AD dementia	2.0	0.0	1.0E-12
Typical AD	2.0	0.0	1.0E-12
Atypical AD	1.0	0.0	1.0E-12
Mixed AD	2.0	0.0	1.0E-12
Preclinical states of AD	1.502	0.5	0.00458
Alzheimer's Pathology	2.0	0.0	1.0E-12
Mild Cognitive Impairment	1.0	0.0	1.0E-12

**Table 6:** **The total probability value for Alzheimer's disease presence due to alterations in *miRNAs* biomarker of the patient**.

**Alzheimer's disease classification**	**Probability of Alzheimer's disease presence (in response to miRNAs biomarker)**
Prodromal AD	0.562
AD dementia	1.0
Typical AD	1.0
Atypical AD	0.0
Mixed AD	1.0
Preclinical states of AD	0.502
Alzheimer's Pathology	1.0
Mild Cognitive Impairment	0.0

*The results revealed the highest probability 1 for the case of AD Pathology, while Prodromal AD and Mixed AD show also high scores*.

Thus, importing the adjusted data below to the Winbugs, in the case of *ADPathology* given that the miRNAs' variable is “True”, the exported probability is 1.

**Data List**

(Age_Inheritance =2, MetalIons=2, APP=2, Cytokines=2, DailyActivities=2, p53=2,

p.Age_Inheritance = c(0.99,0.01),

p.Ab = structure(.Data = c(0.50,0.50,0.50,0.50,0.50,0.50,0.50,0.50), .Dim = c(2,2,2)),

p.Tau_Phospho =structure(.Data = c(1,0,1,0), .Dim = c(2,2)),

p.MetalIons = c(0.76, 0.24),

p.LewyBodies=structure(.Data = c(0.884,0.116,0.884,0.116), .Dim = c(2,2)),

p.Hypertension=structure(.Data = c(1,0,1,0), .Dim = c(2,2)),

p.Depression=structure(.Data = c(1,0,1,0), .Dim = c(2,2)),

p.Smoking=structure(.Data = c(1,0,1,0), .Dim = c(2,2)),

p.Diabetes=structure(.Data = c(1,0,1,0), .Dim = c(2,2)),

p.Obesity=structure(.Data = c(1,0,1,0), .Dim = c(2,2)),

p.PhysicalActivity=structure(.Data = c(1,0,1,0), .Dim = c(2,2)),

p.APP = c(0.90,0.10),

p.GTP = structure(.Data = c(1,0,1,0), .Dim = c(2,2)),

p.APOE4 = structure(.Data = c(1,0,1,0), .Dim = c(2,2)),

p.PS1_2 = structure(.Data = c(1,0,1,0), .Dim = c(2,2)),

p.Cytokines = c(1,0),

p.SenilePlaques = structure(.Data = c(1,0,1,0,1,0,1,0), .Dim = c(2,2,2)),

p.Unbalance_Ca = structure(.Data = c(1,0,1,0,1,0,1,0), .Dim = c(2,2,2)),

p.Vascular = structure(.Data = c(0,1,0,1,0,1,0,1), .Dim = c(2,2,2)),

p.LogopenicAphasiaCortexAtrophy =structure(.Data = c(1,0,1,0), .Dim = c(2,2)),

p.MemoryHippocampalLoss = structure(.Data = c(1,0,1,0), .Dim = c(2,2)),

p.ExecLangPrax=structure(.Data = c(1,0,1,0), .Dim = c(2,2)),

p.VisualNeuropsychiatric =structure(.Data = c(1,0,1,0), .Dim = c(2,2)),

p.DailyActivities = c(0,1),

p.OxidStress1=structure(.Data = c(1,0,1,0,1,0,1,0), .Dim = c(2,2,2)),

p.OxidStress2=structure(.Data = c(1,0,1,0,1,0,1,0), .Dim = c(2,2,2)),

p.Inflamation1=structure(.Data = c(1,0,1,0,1,0,1,0), .Dim = c(2,2,2)),

p.Inflamation2=structure(.Data = c(1,0,1,0,1,0,1,0), .Dim = c(2,2,2)),

p.Isoprostanes1=structure(.Data = c(1,0,1,0,1,0,1,0), .Dim = c(2,2,2)),

p.Isoprostanes2=structure(.Data = c(1,0,1,0,1,0,1,0), .Dim = c(2,2,2)),

p.Mito1=structure(.Data = c(1,0,1,0,1,0,1,0), .Dim = c(2,2,2)),

p.Mito2=structure(.Data = c(1,0,1,0,1,0,1,0), .Dim = c(2,2,2)),

p.Mito3=structure(.Data = c(1,0,1,0), .Dim = c(2,2)),

p.MFN1 = structure(.Data = c(1,0,1,0), .Dim = c(2,2)),

p.OPA1= structure(.Data = c(1,0,1,0), .Dim = c(2,2)),

p.DVLP= structure(.Data = c(1,0,1,0), .Dim = c(2,2)),

p.FIS1= structure(.Data = c(1,0,1,0), .Dim = c(2,2)),

p.p53 = c(0.75,0.25),

p.Ab_APP= structure(.Data = c(1,0,1,0), .Dim = c(2,2)),

p.miRNAs=structure(.Data=c(0,1,0,1), .Dim = c(2,2)),

p.MCI_due_to_DayLiving=structure(.Data=c(1,0,1,0), .Dim=c(2,2)))

After 10000 iterations, the mean value of *ADPathology* is calculated as:

$$\begin{array}{l} EADPathology=2\;*\;p_{ADPathology}+1.\left(1-p_{ADPathology}\right)=2,\\ P\left(ADPathology|miRNAs\right)=\;p_{ADPathology}=2-1=1. \end{array}$$

### Example 3

In the third example, without the age being a risk factor (<60) the most common case is presented, where both Amyloid-beta and Tau proteins' abnormalities occur, with additional ‘True’ values in the Age_Inheritance, APP, APOE4 and Vascular variables of the probabilistic model (Figure 5).

**Data List**

(Age_Inheritance =2, MetalIons=2, APP=2, Cytokines=2, DailyActivities=2, p53=2,

p.Age_Inheritance = c(0.57,0.43),

p.Ab= structure(.Data = c(0,1,0,1,0,1,0,1), .Dim = c(2,2,2)),

p.Tau_Phospho =structure(.Data = c(0,1,0,1), .Dim = c(2,2)),

p.MetalIons = c(0.76, 0.24),

p.LewyBodies=structure(.Data = c(0.884,0.116,0.884,0.116), .Dim = c(2,2)),

p.Hypertension=structure(.Data = c(1,0,1,0), .Dim = c(2,2)),

p.Depression=structure(.Data = c(1,0,1,0), .Dim = c(2,2)),

p.Smoking=structure(.Data = c(1,0,1,0), .Dim = c(2,2)),

p.Diabetes=structure(.Data = c(1,0,1,0), .Dim = c(2,2)),

p.Obesity=structure(.Data = c(1,0,1,0), .Dim = c(2,2)),

p.PhysicalActivity=structure(.Data = c(1,0,1,0), .Dim = c(2,2)),

p.APP = c(0.50,0.50),

p.GTP = structure(.Data = c(1,0,1,0), .Dim = c(2,2)),

p.APOE4 = structure(.Data = c(0.30,0.70,0.30,0.70), .Dim = c(2,2)),

p.PS1_2 = structure(.Data = c(1,0,1,0), .Dim = c(2,2)),

p.Cytokines = c(1,0),

p.SenilePlaques = structure(.Data = c(1,0,1,0,1,0,1,0), .Dim = c(2,2,2)),

p.Unbalance_Ca = structure(.Data = c(1,0,1,0,1,0,1,0), .Dim = c(2,2,2)),

p.Vascular = structure(.Data = c(0,1,0,1,0,1,0,1), .Dim = c(2,2,2)),

p.LogopenicAphasiaCortexAtrophy =structure(.Data = c(1,0,1,0), .Dim = c(2,2)),

p.MemoryHippocampalLoss = structure(.Data = c(1,0,1,0), .Dim = c(2,2)),

p.ExecLangPrax=structure(.Data = c(1,0,1,0), .Dim = c(2,2)),

p.VisualNeuropsychiatric =structure(.Data = c(1,0,1,0), .Dim = c(2,2)),

p.DailyActivities = c(0,1),

p.OxidStress1=structure(.Data = c(1,0,1,0,1,0,1,0), .Dim = c(2,2,2)),

p.OxidStress2=structure(.Data = c(1,0,1,0,1,0,1,0), .Dim = c(2,2,2)),

p.Inflamation1=structure(.Data = c(1,0,1,0,1,0,1,0), .Dim = c(2,2,2)),

p.Inflamation2=structure(.Data = c(1,0,1,0,1,0,1,0), .Dim = c(2,2,2)),

p.Isoprostanes1=structure(.Data = c(1,0,1,0,1,0,1,0), .Dim = c(2,2,2)),

p.Isoprostanes2=structure(.Data = c(1,0,1,0,1,0,1,0), .Dim = c(2,2,2)),

p.Mito1=structure(.Data = c(1,0,1,0,1,0,1,0), .Dim = c(2,2,2)),

p.Mito2=structure(.Data = c(1,0,1,0,1,0,1,0), .Dim = c(2,2,2)),

p.Mito3=structure(.Data = c(1,0,1,0), .Dim = c(2,2)),

p.MFN1 = structure(.Data = c(1,0,1,0), .Dim = c(2,2)),

p.OPA1= structure(.Data = c(1,0,1,0), .Dim = c(2,2)),

p.DVLP= structure(.Data = c(1,0,1,0), .Dim = c(2,2)),

p.FIS1= structure(.Data = c(1,0,1,0), .Dim = c(2,2)),

p.p53 = c(0.75,0.25),

p.Ab_APP= structure(.Data = c(0,1,0,1), .Dim = c(2,2)),

p.miRNAs=structure(.Data=c(0,1,0,1), .Dim = c(2,2)),

p.MCI_due_to_DayLiving=structure(.Data=c(1,0,1,0), .Dim = c(2,2)))

Given the initial data set above, after 10000 iterations the estimated probabilities of the eight AD categories (Tables 7, 8) reveals high risk for AD presence. The results highlight the role of Amyloid-beta and Tau proteins and emphasize their importance and effectiveness in AD aggravation.

**Table 7:** **WINBUGS statistics for Alzheimer's disease categories according to Example 3**.

**Node**	**Mean**	**Standard deviation**	**MC error after 10000 iterations in WinBugs**
Prodromal AD	2.0	0.0	1.0E-12
AD dementia	2.0	0.0	1.0E-12
Typical AD	2.0	0.0	1.0E-12
Atypical AD	1.0	0.0	1.0E-12
Mixed AD	2.0	0.0	1.0E-12
Preclinical states of AD	2.0	0.0	1.0E-12
Alzheimer's Pathology	1.0	0.0	1.0E-12
Mild Cognitive Impairment	1.0	0.0	1.0E-12

**Table 8:** **The total probability value for Alzheimer's disease presence due to alterations in Ab, Tau/TotalTau, age/inheritance, APP, APOE4 and Vascular disorders of the patient**.

**Alzheimer's disease classification**	**Probability of Alzheimer's disease presence (in response to Ab, Tau/TotalTau, age/inheritance, APP, APOE4 and Vascular disorders biomarkers)**
Prodromal AD	1.0
AD dementia	1.0
Typical AD	1.0
Atypical AD	0.0
Mixed AD	1.0
Preclinical states of AD	1.0
Alzheimer's Pathology	0.0
Mild Cognitive Impairment	0.0

*As it expected, the results revealed high probabilities for the cases of Prodromal AD, AD dementia, Typical AD, Mixed AD, Preclinical states of AD*.

### Example 4

In the fourth example, the hypothetical patient (age <60) is a Smoker with an Obesity problem and Depression symptoms (Figure 6). The Bayesian model calculates the probabilities respectively,

$$\begin{array}{l} P\left(ProdromalAD|Depression,\;Obesity,\;Smoking\right)\\ and\;P\left(MixedAD|Depression,\;Obesity,\;Smoking\right).\\ P\left(ProdromalAD|Depression,Obesity,\;Smoking\right)\\ =P\left(MixedAD|Depression,Obesity,\;Smoking\right)=\;\\ =\frac{\begin{array}{c} P\left(ProdromalAD,MixedAD|Depression,Obesity,\;Smoking\right)\\ P\left(ProdromalAD,MixedAD\right) \end{array}}{P\left(Depression,Obesity,\;Smoking\right)}\\ =0.464. \end{array}$$

**Data List**

(Age_Inheritance =2, MetalIons=2, APP=2, Cytokines=2, DailyActivities=2, p53=2,

p.Age_Inheritance = c(0.57,0.43),

p.Ab= structure(.Data = c(1,0,1,0,1,0,1,0), .Dim = c(2,2,2)),

p.Tau_Phospho =structure(.Data = c(1,0,1,0), .Dim = c(2,2)),

p.MetalIons = c(0.76, 0.24),

p.LewyBodies=structure(.Data = c(0.884,0.116,0.884,0.116), .Dim = c(2,2)),

p.Hypertension=structure(.Data = c(1,0,1,0), .Dim = c(2,2)),

p.Depression=structure(.Data = c(0.868,0.132,0.868,0.132), .Dim = c(2,2)),

p.Smoking=structure(.Data = c(0.726,0.274,0.726,0.274), .Dim = c(2,2)),

p.Diabetes=structure(.Data = c(1,0,1,0), .Dim = c(2,2)),

p.Obesity=structure(.Data = c(0.966,0.034,0.966,0.034), .Dim = c(2,2)),

p.PhysicalActivity=structure(.Data = c(1,0,1,0), .Dim = c(2,2)),

p.APP = c(0.50,0.50),

p.GTP = structure(.Data = c(1,0,1,0), .Dim = c(2,2)),

p.APOE4 = structure(.Data = c(1,0,1,0), .Dim = c(2,2)),

p.PS1_2 = structure(.Data = c(1,0,1,0), .Dim = c(2,2)),

p.Cytokines = c(1,0),

p.SenilePlaques = structure(.Data = c(1,0,1,0,1,0,1,0), .Dim = c(2,2,2)),

p.Unbalance_Ca = structure(.Data = c(1,0,1,0,1,0,1,0), .Dim = c(2,2,2)),

p.Vascular = structure(.Data = c(0,1,0,1,0,1,0,1), .Dim = c(2,2,2)),

p.LogopenicAphasiaCortexAtrophy =structure(.Data = c(1,0,1,0), .Dim = c(2,2)),

p.MemoryHippocampalLoss = structure(.Data = c(1,0,1,0), .Dim = c(2,2)),

p.ExecLangPrax=structure(.Data = c(1,0,1,0), .Dim = c(2,2)),

p.VisualNeuropsychiatric =structure(.Data = c(1,0,1,0), .Dim = c(2,2)),

p.DailyActivities = c(1,0),

p.OxidStress1=structure(.Data = c(1,0,1,0,1,0,1,0), .Dim = c(2,2,2)),

p.OxidStress2=structure(.Data = c(1,0,1,0,1,0,1,0), .Dim = c(2,2,2)),

p.Inflamation1=structure(.Data = c(1,0,1,0,1,0,1,0), .Dim = c(2,2,2)),

p.Inflamation2=structure(.Data = c(1,0,1,0,1,0,1,0), .Dim = c(2,2,2)),

p.Isoprostanes1=structure(.Data = c(1,0,1,0,1,0,1,0), .Dim = c(2,2,2)),

p.Isoprostanes2=structure(.Data = c(1,0,1,0,1,0,1,0), .Dim = c(2,2,2)),

p.Mito1=structure(.Data = c(1,0,1,0,1,0,1,0), .Dim = c(2,2,2)),

p.Mito2=structure(.Data = c(1,0,1,0,1,0,1,0), .Dim = c(2,2,2)),

p.Mito3=structure(.Data = c(1,0,1,0), .Dim = c(2,2)),

p.MFN1 = structure(.Data = c(1,0,1,0), .Dim = c(2,2)),

p.OPA1= structure(.Data = c(1,0,1,0), .Dim = c(2,2)),

p.DVLP= structure(.Data = c(1,0,1,0), .Dim = c(2,2)),

p.FIS1= structure(.Data = c(1,0,1,0), .Dim = c(2,2)),

p.p53 = c(0.75,0.25),

p.Ab_APP= structure(.Data = c(1,0,1,0), .Dim = c(2,2)),

p.miRNAs=structure(.Data=c(0,1,0,1), .Dim = c(2,2)),

p.MCI_due_to_DayLiving=structure(.Data=c(1,0,1,0), .Dim = c(2,2)))

Given the initial dataset above, after 10000 iterations the estimated probabilities of the eight AD categories (Tables 9, 10) reveals a medium risk for AD presence due Depression, Smoking and Obesity and a set of risk factors for related comorbidities. The results in general, highlight the role of Hypertension, Depression, Smoking, Diabetes, Obesity, and Physical Inactivity as potential AD biomarkers and emphasize their importance and effectiveness in AD aggravation. The calculated probabilities verify the latest clinical findings (Modrego and Ferrández, 2004; Barnes and Yaffe, 2011) where the combination of Mild Cognitive Impairment and Depression in patients, doubles the risk of Alzheimer Dementia development compared with those without depression.

**Table 9:** **WINBUGS statistics for Alzheimer's disease categories according to Example 4**.

**Node**	**Mean**	**Standard deviation**	**MC error after 10000 iterations in WinBugs**
Prodromal AD	1.464	0.4987	0.004383
AD dementia	1.0	0.0	1.0E-12
Typical AD	1.0	0.0	1.0E-12
Atypical AD	1.0	0.0	1.0E-12
Mixed AD	1.464	0.4987	0.004383
Preclinical states of AD	1.0	0.0	1.0E-12
Alzheimer's Pathology	1.0	0.0	1.0E-12
Mild Cognitive Impairment	1.0	0.0	1.0E-12

**Table 10:** **The total probability value for Alzheimer's disease presence due to Obesity and Depression problems in a smoker patient**.

**Alzheimer's disease classification**	**Probability of Alzheimer's disease presence (in response to Depression, Smoking and Obesity biomarkers)**
Prodromal AD	0.464
AD dementia	0.0
Typical AD	0.0
Atypical AD	0.0
Mixed AD	0.464
Preclinical states of AD	0.0
Alzheimer's Pathology	0.0
Mild Cognitive Impairment	0.0

*The results revealed medium probabilities for the cases of Prodromal AD and Mixed AD*.

## Discussion

While AD is a hardly curable disease, few computational diagnostic tools have been published during the last years, for the evaluation of biomarkers and symptoms and the automated prediction of the disease. There are algorithms for an automated Dementia identification based on MRI, PET and SPECT imaging analysis using Bayes classifiers, support vector machines, and artificial neural networks (Zheng et al., 2016). According to these specific methods, the systems have to be trained with as many cases as possible to improve accuracy in a clinical dataset. There is also a tool for the automatic diagnosis of AD via the combination of PET Images and Neuropsychological Test Data (Segovia et al., 2014). According to its documentation, authors using a multi-kernel classification approach trained a mixed data set to improve the accuracy of their diagnosis in compare with other methods that evaluate imaging results exclusively. It is important to mention another latest clinical decision support system for AD that combines a Rule-Based System with a Clinical Guideline-Based System, and it is modeled through a Bayesian Network (Seixas et al., 2014). This is another case of a decision trained system that accesses a specific dataset of biomarkers to provide an accurate diagnosis of Dementia, Alzheimer's and MCI.

In the current method, all the known AD biomarkers are combined in a complex Bayesian Network to establish a medical diagnostic decision system for AD, not as a generic diagnostic result but mainly as a more sophisticated probabilistic outcome referred to all the eight categories of AD classification. The proposed statistical model is multi-parametric, targeting the convergence of several independent data like plasma and CSF tests with behavioral or imaging tests and their representation through prior categorical distributions. The proposed AD Bayesian model uses the WinBUGS 1.4.3 software, and all the experiments have been executed in a personal computer with medium performance. While the WinBUGS program cannot be used as an online software, a friendly website (http://alzheimers.edu.gr) has also been designed for individual users and medical staff, for the submission and analysis of anonymous AD tests results. External users can upload biomarkers' results in the form of “True” or “False” and receive the personalized exported statistics in their email account. Medical staff can use the prognostic tool even for individual cases, having in mind that in the Bayesian Inference thousands of sample iterations are automatically executed to pre-define the unknown prior distribution of the model and calculate the posterior distribution of the heterogeneous data with high accuracy. Since the proposed probabilistic model is based on conditional probabilities, it must be noted that the calculated error is only the Monte Carlo Error that measures the variability of each estimation due to simulation, increasing the accuracy of the model almost to the 100%. Besides the categorical values, the medical staff is prompted to upload in the webpage, the analytic test results, any medications or other special conditions that refer to the under consideration patient, anonymously or even more to ask for an upgrade of the model, if new dynamic relations occur between the biomarkers, or new biomarkers being identified. The authors of this computational method are in the process of designing, organizing and implement an open biological database for the data sharing of biomarkers assessment (Frasier, 2016), the dissemination of accurate clinical practices and the validation of the current method. In this way, we could replace in the future the categorical values of the current model with real datasets from observational studies improving the cooperation between scientists, targeting a holistic solution against AD. Including a large set of multilevel biomarkers, the proposed diagnostic method has not been validated yet. Therefore we will ask and embed in our system every time, the final diagnosis of the clinicians as a feedback for the evaluation and improvement of our model.

We strongly believe and work in this direction, that an international open biological database for hosting AD clinical results, could benefit the research against the disease helping scientists to re-evaluate their diagnostic models and treatments or even more consider alternative solutions.

Apparently, the proposed Bayesian approach can be extended to several other related neurodegenerative disorders where the early recognition of symptoms is a crucial factor for an efficient treatment procedure and in similar cases of unknown etiology such as the hypothesis of Developmental Origins of Health and Disease and the research on epigenetic mechanisms in epidemiological studies (Barker and Osmond, 1986; Barker et al., 1989, 1993).

## Author contributions

AA study concept and design, analysis and interpretation of data, study supervision, preparation of the final manuscript, critical revision of manuscript for intellectual content. VM study concept and design, acquisition of data, analysis and interpretation of data, writing of the first draft. NG critical revision of manuscript for intellectual content. MK critical revision of manuscript for intellectual content.

## Funding

This study is based on work that has been supported by the AFnP Engineering, Chemicals and Consumables GmbH. AA received grant from AFnP Engineering, Chemicals and Consumables GmbH; VM has nothing to disclose; NG has nothing to disclose; MK has nothing to disclose.

### Conflict of interest statement

The authors declare that the research was conducted in the absence of any commercial or financial relationships that could be construed as a potential conflict of interest.
